# Micronutrient and Trace Element Levels in Serum of Women With Uterine Fibroids in Lagos

**DOI:** 10.7759/cureus.18638

**Published:** 2021-10-10

**Authors:** Christian C Makwe, Adaiah P Soibi-Harry, Garba S Rimi, Okechukwu A Ugwu, Abisoye T Ajayi, Taiwo A Adesina, Kehinde S Okunade, Ayodeji A Oluwole, Rose I Anorlu

**Affiliations:** 1 Obstetrics and Gynaecology, Lagos University Teaching Hospital/College of Medicine, University of Lagos, Lagos, NGA; 2 Obstetrics and Gynaecology, Lagos University Teaching Hospital, Lagos, NGA; 3 Department of Radiodiagnosis, Lagos University Teaching Hospital, Lagos, NGA; 4 Obstetrics and Gynaecology, College of Medicine, University of Lagos, Lagos, NGA

**Keywords:** calcium, vitamin d, trace elements, uterine fibroids, micronutrients

## Abstract

Background: Uterine fibroids significantly affect the quality of life of reproductive-age women. The socioeconomic cost and psychological strain on patients cannot be overemphasized. The role of diet and micronutrients on the onset and development of uterine fibroids has come under review in recent times. This study assessed the levels of some micronutrients and trace elements in the serum of women with uterine fibroids.

Methods: Eighty-eight women were recruited from the Gynecology Outpatient Clinic of Lagos University Teaching Hospital, 44 with uterine fibroids and 44 women without uterine fibroids. Blood samples were obtained and analyzed for serum levels of selected micronutrients (vitamins A, C, D, and E) and trace elements (calcium, magnesium, and phosphorus). Pelvic ultrasonography was performed on all study participants.

Results: Women with uterine fibroids had statistically significant lower serum levels of vitamin C (1.20 ± 0.59 vs 1.62 ± 1.75 mg/dl; p = 0.01), vitamin D (34.23 ±10.67 vs 37.06 ±11.46 ng/ml; p = 0.04), and calcium (2.27 ± 0.19 vs 2.32 ± 0.09 mmol/L; p = 0.02) compared with women without uterine fibroids. There was no significant difference in the serum levels of vitamins A (39.63 ± 15.71 vs 40.09 ±15.26 μ/dl; p = 0.91), vitamin E (5.44 ± 4.65 vs 5.26 ± 4.62 µg/mL; p = 0.87), magnesium (0.89 ± 0.09 vs 0.89 ± 0.08 mmol/L; p = 0.78), and phosphorus (1.29 ± 0.38 vs 1.19 ± 0.17 mmol/L; p = 0.14) in women with uterine fibroids compared to those without uterine fibroids.

Conclusion: This study showed lower serum levels of vitamin C, vitamin D, and calcium in women with uterine fibroids when compared to women without uterine fibroids. It is possible that these micronutrients and trace elements may play a role in the etiopathogenesis, progression, and/or proliferation of uterine fibroids. However, whether the findings of low serum levels of these elements are a cause or an effect of uterine fibroid, is yet to be determined.

## Introduction

Uterine fibroids are benign gynecological tumors seen in reproductive-age women and the most common indication for hysterectomy globally [1]. Women with symptomatic uterine fibroids present with a history of heavy menstrual bleeding, abnormal uterine bleeding, pelvic pressure symptoms like urinary frequency, incontinence, constipation, and tenesmus and pelvic pain. It is indeed a disease of public health importance as its impact on the quality of life, loss of work hours, short-term disability, absenteeism and psychological strain on patients cannot be overemphasized. In fact, a study by Cardozo et al. found the annual societal cost of uterine fibroid in the United States to be more than that for breast, colon, or ovarian cancer, and nearly one-fifth the cost of diabetes [2].

The importance of micronutrients is expressed in the characteristic deficiency states that arise when these nutrients are deficient in the human body. Most notable are scurvy from vitamin C deficiency, rickets from vitamin D deficiency, megaloblastic anaemia from B12 deficiency, osteoporosis from calcium deficiency, etc. Research into the role of micronutrients in the prevention or treatment of disease as well as optimizing health has been on the increase as more people seek natural alternatives and scientists better understand the biochemistry of these substances. Some epidemiological studies suggest a potential association between diet and hormone-modulated diseases [3,4]. Vitamins and minerals, collectively known as micronutrients play very important roles in metabolism and tissue maintenance, with most of them functioning as cofactors, coenzymes, gene control, and antioxidants. As cofactors, they modulate the activity of enzymes or are an integral part of the enzyme prosthetic group; as coenzymes, they play an active role in the complexing of biochemical reactions, in gene control they regulate transcription of receptors, and as antioxidants, they act as scavengers of free radicals [5]. Vitamins are available either as water-soluble (vitamins B and C) or fat-soluble (vitamins A, D, E, and K) while minerals are available as microminerals (iron, copper, iodine, zinc, and fluoride) or trace elements (calcium, magnesium, phosphorus, sodium, and potassium). Micronutrients are not produced by the body and must therefore be obtained from dietary sources.

Although the etiopathogenesis of uterine fibroids is not well understood, it is known, that proliferation of smooth muscle cells and excessive extracellular matrix deposition are the main features of uterine fibroids development and that oestrogen and progesterone play a prominent role in the initiation and promotion of uterine fibroids [6-8]. The relationship between breast cancer (a disease modulated by oestrogen) and micronutrients suggests that women who ate diets rich in micronutrients had a reduced risk of developing breast cancer [9,10]. Islam et al. suggested that certain dietary or alternative treatments may be effective for uterine fibroids [11]. Several observational studies also suggest that increased consumption of fruits, vegetables, and dairy may reduce the risk of developing uterine fibroids [12,13].

Micronutrients have certain properties that may reduce the risk of uterine fibroids in women, for example, carotenoids. Vitamins C and E have antioxidant properties that stabilize cell membranes and prevent DNA damage and consequent mutations as a result of oxidative stress. Magnesium, calcium, and phosphorus function as co-factors in the synthesis and repair of DNA and regulation of hormones [14]. Ciebiera et al. found elevated levels of alpha-tocopherol in women with uterine fibroid and concluded that vitamin E as a phytoestrogen may modulate estrogen receptors and increase oestrogen levels, thereby increasing the odds of developing uterine fibroids [15]. The few studies that have evaluated the role of micronutrients in the pathogenesis of uterine fibroids have been inconsistent and carried out mostly in the Caucasian and African-American populations as against African women in whom the disease burden is greater. This study, therefore, seeks to compare the pre-operative serum levels of some micronutrients (vitamins A, C, D, and E) and trace elements (calcium, magnesium, and phosphorus) in women with and without uterine fibroids in Lagos.

## Materials and methods

This was a cross-sectional study carried out at the Gynaecology Outpatient Clinic of Lagos University Teaching Hospital from July 1, 2019, to January 31, 2020. Eighty-eight women aged 20-45 years who met the eligibility criteria and gave a written informed consent were recruited into the study. A structured proforma was used to get information by face-to-face interview. Data collected included: socio-demographic parameters, medical, drug and obstetric history, menstrual history, history of dysmenorrhea, deep dyspareunia, non-cyclical chronic pelvic pain, gastrointestinal and lower urinary tract symptoms, and history of uterine fibroids in first degree relatives. Anthropometry was assessed and body mass index (BMI) was calculated.

Women who were pregnant, lactating, smokers, currently using vitamin supplements were excluded from the study to reduce confounders.

At enrolment, study participants were screened using a portable 2D ultrasound unit Sonostar 15 SS-10 (Sonostar Technologies Co., Ltd., Guangdong, China) with a 5 MHz probe at the Gynaecology Outpatient Clinic by a single investigator (ASH). Eligible participants were then referred to the Department of Radiodiagnosis LUTH for a pelvic ultrasonography with Doppler studies by an experienced sonologist (ATA). The pelvic ultrasonography was performed using a 3D ultrasound unit TUS-X100S (Toshiba Diagnostic Ultrasound Systems Ltd., Japan) with a transabdominal (5.0 MHz) and/or transvaginal (7.0 MHz) ultrasound probes as appropriate. Pelvic scan was interpreted in real-time and images stored in soft and hard copies. Each fibroid was measured in sagittal and axial views and the maximum diameter was taken for the study. Women with ultrasound diagnosis of uterine fibroids were assigned to the fibroid group (n=44) and those without uterine fibroids were age matched within (±2 years) to those with uterine fibroids and assigned to the no fibroid group (n=44).

Sample collection and laboratory methods

A plain vacutainer and an ethylenediaminetetraacetic acid (EDTA) vacutainer were labelled with the unique identification number of each woman. Ten milliliters of venous blood was obtained from the antecubital vein, using a peripheral venous catheter, 5 ml was placed in a plain vacutainer and 5 ml into an EDTA vacutainer. The samples were allowed to clot for two hours at room temperature, centrifuged using Eppendorf 5415C centrifuge (Eppendorf AG, Germany) at 3000 rpm for 15 minutes, the serum supernatant removed with a sterile Pasteur pipette, and equal volumes were aliquoted into two cryogenic vials and frozen at −80 °C at the Central Research Laboratory in LUTH. The serum samples were allowed to thaw on the workbench and all assays performed at room temperature as one batch. 

Assay of Vitamin A

Determination of vitamin A was performed using human vitamin A Elisa Kit, CSB-E07889h (Cusabio Technology LLC, Houston, TX, USA), following the manufacturer’s instruction.

Assay of Vitamin C

Determination of Vitamin C was performed using human vitamin C Elisa Kit, MBS726748 (MyBioSource, Inc. San Diego, CA, USA), following the manufacturer’s instruction.

Assay of Vitamin D

Determination of Vitamin D was performed using human 25 (OH) vitamin D Elisa Kit, VD220B (Calbiotech, Inc., El Cajon, CA, USA), following the manufacturer’s instruction.

Assay of Vitamin E

Determination of Vitamin E was performed using human vitamin E Elisa Kit, CSB-E07893h (Cusabio Technology LLC, Houston, TX, USA), following the manufacturer’s instruction.

The optical density of each micronutrient was determined using a microplate reader MR-96A-Mindray (China) set to 450 nm.

Assay of Calcium, Magnesium, and Phosphorus

Serum calcium, magnesium, and phosphorus levels were analysed using a fully automated chemistry analyzer, COBAS INTEGRA 400 (Roche Diagnostics International AG, Rotkreuz, Switzerland) plus by using system compatible packs.

Statistical analysis

Analysis was done using the IBM Statistical Package for Social Sciences (SPSS) Statistics for Windows, Version 25.0 (Armonk, NY: IBM Corp). Data were presented as tables. Shapiro-Wilk test was used to test for normality. Normally distributed continuous variables were presented as mean ± standard deviation, while non-parametric variables were presented as median ± interquartile range. Student’s T-test and Chi square test were used to compare normally distributed continuous variables and categorical variables, respectively. P-value less than 0.05 was considered statistically significant.

Ethical considerations

Ethical approval was obtained from the HREC of LUTH, and all included study participants gave voluntary and informed consent. The Helsinki declaration ethical principles were observed throughout the conduct of the study.

## Results

All 88 women, 44 with uterine fibroids and 44 without uterine fibroids were included in the statistical analysis.

Sociodemographic parameters of participants

Table 1 shows the sociodemographic parameters of the two groups. There was no statistically significant difference in the sociodemographic parameters between the two groups.

**Table 1:** Sociodemographic characteristics of participants *Independent t-test; SD: standard deviation.

Variables	Fibroid (N = 44)	No fibroid (N = 44)	χ^2^	p-value
	Mean (SD)	Mean (SD)		
Age	38.3 (6.5)	35.9 (7.5)	t = 1.55	0.132*
Parity	0.93 (1.5)	0.77 (1.3)	t = 0.54	0.592*
BMI (kg/m^2^)	26.88 (5.18)	26.14 (4.38)	t = 0.76	0.470*
	n (%)	n (%)		
Ethnicity				
Igbo	11 (25)	22 (50)	7.94	0.064
Yoruba	22 (50)	18 (40.9)		
Others	11 (25)	4 (9.1)		
Level of education				
Primary	1 (2.3)	1 (2.3)	0.00	1.000
Secondary	7 (15.9)	7 (15.9)		
Tertiary	36 (81.8)	36 (81.8)		
Employment status				
Employed	36 (81.8)	32 (72.7)	2.92	0.234
Unemployed	8 (18.2)	12 (27.3)		
Marital status				
Married	26 (59.1)	26 (59.1)	0.00	1.000
Single	18 (40.9)	18 (40.9)		
Socioeconomic status				
Semi-skilled	15 (34.1)	20 (45.5)	1.41	0.493
Skilled professional	24 (54.5)	21 (47.7)		
Unskilled	5 (11.4)	3 (6.8)		

Baseline characteristics of participants

Table 2 shows the baseline characteristics of the participants. More women with uterine fibroids reported dysmenorrhea (84.1% vs 47.7%; p < 0.001), heavy menstrual bleeding (72.7% vs 40.9%; p = 0.003), urinary frequency (63.6% vs 18.2%; p < 0.001), use of analgesics (70.5% vs 31.8%; p < 0.001), and presence of fibroids in their first-degree relatives (38.6% vs 18.2%; p = 0.034) when compared with women without uterine fibroids.

**Table 2:** Baseline characteristics of participants

Variables	Fibroid (n = 44)	No fibroid (n = 44)	χ^2^	p-value
	n (%)	n (%)		
Heavy menstrual bleeding				
Yes	32 (72.7)	18 (40.9)	9.08	0.003
No	12 (27.3)	26 (59.1)		
Dysmenorrhoea				
Yes	37 (84.1)	21 (47.7)	12.95	< 0.001
No	7 (15.9)	23 (52.3)		
Dyspareunia				
Yes	9 (20.5)	10 (22.7)	0.07	0.802
No	35 (79.5)	34 (77.3)		
Analgesic use				
Yes	31 (70.5)	14 (31.8)	13.14	< 0.001
No	13 (29.5)	30 (68.2)		
Recent blood transfusion				
Yes	9 (20.5)	3 (6.8)	3.47	0.064
No	35 (79.5)	41 (93.2)		
Urinary frequency				
Yes	28 (63.6)	8 (18.2)	18.80	< 0.001
No	16 (36.4)	36 (81.8)		
Fibroid in the first-degree relative				
Yes	17 (38.6)	8 (18.2)	4.53	0.034
No	27 (61.4)	36 (81.8)		

Ultrasonographic parameters of uterine fibroids

Table 3 shows the ultrasonographic findings among participants with uterine fibroids. About half (47.7%) of the participants had uterine fibroids less than 20 weeks’ gestational size. Half of the fibroids were intramural in position.

**Table 3:** Ultrasonographic parameters of uterine fibroids ∞Abdominal examination in comparison with single intrauterine gestation

Parameters	N=44
	n (%)
Uterine size in weeks^∞^	
<20 weeks	29 (65.9)
>20 weeks	15 (34.1)
Location of fibroids	
Type 1 (sub-mucous)	8 (18.2)
Type 2–5 (intramural)	22 (50)
Type 6 (sub-serous)	7 (15.9)
Hybrid	7 (15.9)
Number of fibroids	
Solitary	13 (29.5)
Multiple	31 (70.5)
Maximum diameter of largest fibroids	
<50 mm	9 (20.5)
50–100 mm	21 (47.7)
>100 mm	14 (31.8)

Biochemical characteristics of participants

Table 4 shows the biochemical characteristics of participants in both groups. Women with uterine fibroids had statistically significantly lower serum levels of vitamin C, vitamin D, and calcium when compared with women without uterine fibroids. There was no significant difference in the serum levels of vitamins A, vitamin E, magnesium, and phosphorus in women with uterine fibroids when compared with those without uterine fibroids.

**Table 4:** Biochemical characteristics of participants

Variables	Fibroid (n=44) M (SD)	No fibroid (n=44) M (SD)	t	p-value	Normal range
Serum vitamin A (μ/dl)	39.63 (15.71)	40.09 (15.26)	0.20	0.91	20–60
Serum vitamin C (mg/dl)	1.20 (0.59)	1.62 (1.75)	−1.07	0.01	0.4–2
Serum vitamin D (ng/ml)	34.23 (10.67)	37.06 (11.46)	−1.20	0.04	25–80
Serum vitamin E (µg/ml)	5.44 (4.65)	5.26 (4.62)	0.23	0.87	5.5–17 µg/mL
Serum phosphorus (mmol/L)	1.29 (0.38)	1.19 (0.17)	1.48	0.14	0.97–1.45 mmol/L
Serum calcium (mmol/L)	2.27(0.19)	2.32 (0.09)	−1.53	0.02	2.25–2.62 mmol/L
Serum magnesium (mmol/L)	0.89(0.09)	0.89 (0.08)	0.11	0.78	0.85–1.10

## Discussion

This study aimed to measure and compare serum levels of some micronutrients (vitamin A, vitamin C, vitamin D, vitamin E, calcium, magnesium, and phosphorus) in women with and without uterine fibroids.

In this study, we did not find any significant difference in the sociodemographic and baseline characteristics between the two groups of women. This study found a statistically significant difference in the serum levels of vitamin C, vitamin D, and calcium between the two groups of women. Women with uterine fibroids had lower levels of vitamin C, vitamin D, and calcium when compared with women without uterine fibroids. On the other hand, there was no significant difference in the serum levels of vitamins A, vitamin E, magnesium, and phosphate between women with uterine fibroids and those without. Interestingly, most women in both study groups had the recommended levels or the reference range of all analyzed micronutrients [16].

The finding of lower vitamin C levels in women with uterine fibroids is consistent with the study of Oyeyemi et al. [17]. This may be due to the similarity in the racial population of the study participants. Vitamin C is well known for its antioxidant properties and some recent epidemiological studies suggest a reduction in levels of antioxidants in uterine fibroids [18,19].

The lower level of vitamin C in women with uterine fibroids may be a cause or an effect relationship. Heinonen et al. in their study concluded that the depletion of vitamin C metabolites may be linked to the large quantity of extracellular matrix in uterine fibroids since vitamin C has been shown to be a cofactor in the synthesis of collagen [20]. But in contrast to the findings of Wise et al. who assessed the association of the dietary intake of fruit, vegetables, and different vitamins including vitamin C, and did not find any significant relationships between vitamin C and uterine fibroids [12].

We also found reduced levels of calcium in women with uterine fibroids, compared to those without fibroid. This is consistent with the findings of Dheeraj and Singh in coastal Odisha, India, but contradicts the findings of Oyeyemi et al. in South-West Nigeria where they found higher levels of calcium in women with uterine fibroids [17,21]. This may be due to the difference in the demographics of the study population.

In addition, our study found lower levels of vitamin D in women with uterine fibroids. This is consistent with several studies that have identified vitamin D deficiency/insufficiency as a risk factor for uterine fibroids [22-24].

Strengths of the study

This was a prospective analytical cross-sectional study carried out amongst black women in Nigeria compared to previous studies. Participants were matched by age, to reduce confounders.

Limitations of the study

This study may not be representative of the general population as it was a hospital-based study.

## Conclusions

Women with uterine fibroids have lower serum levels of vitamin C, vitamin D, and calcium. It is possible that these micronutrients and trace elements may play a role in aetiopathogenesis, progression and or proliferation of uterine fibroids. However, whether the findings of low serum levels of these elements are as a cause or an effect of uterine fibroid, is yet to be determined.
